# A New and Complete Treatise on Gout, and Its Varieties

**Published:** 1840-01

**Authors:** 


Art. II.—*Nuovo Trattato completo della Gotta in genere, e sue specie, cioè Podagra, Chiragra, Gonagra, ed altre Malattie Gottose.* Opera di Giuseppe Ciani, Medico Romano, Dottore in Filosofia e Medicina, &c. Tom. I.—*Roma*, 1838. 8vo, pp. 212.

*A new and complete Treatise on Gout, and its Varieties—Podagra, Chiragra, Gonagra, and on Gouty Diseases in general.* By Joseph Ciani, Physician in Rome, Doctor of Philosophy and Medicine, &c. Vol. I.—*Rome*, 1838.

This is a work which comes into the world at least a century too late: it should have appeared when mere hypotheses, the work of imagination, were received with equal reverence as generalizations of facts, or rational theories legitimately deduced from them. Although it cannot be received as a fair sample of the general mode of philosophizing now prevalent among physicians in Italy, it must be admitted, that its visionary character will make it appear less singular, and cause it to obtain more countenance from learned men in that country than would be the case in England, or even in France or Germany. However, while our own literature is disgraced by the writings of Dr. Ramage and Dr. Dickson, regularly-educated physicians, we are not justified in regarding the absurdities of Dr. Ciani as national, but merely as individual and personal delinquencies.

Dr. Ciani's theory of gout is, he says, altogether new, never having been previously imagined (è del tutto nuova, non fu da alcuno sinora ideata), although it is evidently a spurious breed between the physiological or Broussaian and the modern chemico-medical doctrines. There must be, he says, certain predisposing conditions in gouty subjects before the causes of the disease can take effect; and these conditions are a sanguineous or sanguineo-bilious temperament, a laxity and atony of the muscular system, a plethora *ad vasa* of the venous system of the abdominal viscera, and, lastly, a superabundant accumulation of caloric in the same. If in subjects so predisposed, the digestive processes become disturbed from excessive or too stimulant diet, a superfluous quantity of the phosphate and subphosphate, and also of the carbonate of lime is generated, and not being absorbed from a deficiency of solvents (the phosphates more particularly), they are deposited on the mucous membrane of the gastro-intestinal tube, where, becoming also associated with other heterogeneous principles, they excite an irritation which ends in a true phlogosis of the same membrane, which phlogosis is the more vehement from the previous hypersthenic condition of the affected parts. Sometimes the disease proceeds no further, but remains a circumscribed gouty gastro-enteritis, in which case it constitutes the first variety—*idiopathic gout;* sometimes, the primary inflammation remaining, there is excited, by sympathy, inflammation of the joints, and this is the second variety —*sympathetic gout;* lastly, the primary gastro-enteritis entirely disappearing, is transferred to the joints, or some other part—and this is the *metastatic gout.*

We know not if it will suprise our readers—it certainly did not at all surprise us—to learn, that Dr. Ciani adduces no proof whatever of the presence of his phosphates in the alimentary canal in gout, or of the presence of the primary inflammation said to be excited by it; all is assumption and presumption from begining to end.

The author's therapeutics, as may be supposed, are in due accordance with his theory. The *lactic acid* being the special solvent of the phosphate of lime (the last being only deposited on the intestinal mucous membrane, from defect of the former in the chyme or chyle), becomes, consequently, the special or specific cure of the disease originating in this. "Thus," he says, "eschewing empiricism, and subjecting the gouty to my scientific plan, supported, as it is, by reasoning and confirmed by experience as not merely innocuous but beneficial, we not only need apprehend no sinister result, but may promise a certain cure." But it would be doing injustice to Dr. Ciani, if we did not state that his scientific plan includes much more than the administration of the lactic acid. Under the head of the radical cure of gout, he judiciously prescribes the important hygienic means, of change in the general mode of living, rigorous sobriety, and constant bodily exercise. In the application of his lactic acid, too, for the palliative cure, he requires rigid indications, holding it to be absolutely necessary, before the physician prescribes, that he should be satisfied of the precise cause of the disease in the case before him, that is, whether the gouty matter deposited is the free phosphoric acid itself in excess, the phosphate of lime, or lime by itself. This, however, may seem easier said than done; but Dr. Ciani sees no difficulties in the case, regarding the presence of a sense of heat in the stomach, thirst, flatulence, &c., as indicating the presence of free phosphoric acid, and the absence of these as announcing the existence of the others! We will not trouble our readers with the varieties of the modes of treatment founded on these various indications, but conclude with a brief account of the mode of administering the grand specific—the lactic acid. This is given in the form of lemonade, made by dissolving half a drachm of the acid in eight ounces of water, and sweetening it with six drachms of syrup. This quantity the patient takes every morning fasting, and, again, after supper; and after dinner and supper, he also takes three or four pastilles, prepared with the same acid, and which he allows to dissolve slowly in his mouth. This practice is to be continued, uninterruptedly, for *several years*, with the single exception of omitting the pastilles during the months of July, August, and September, and taking, during their omission, two drachms of cinchona (china), in a little water, before dinner, daily. And thus pleasantly does Dr. Ciani cure his patients, after having pleasantly cured himself of a gout of ten years' standing. We recommend his erudite treatise and his agreeable lemonade to the particular attention of Sir Charles Scudamore, and all other fashionable gout-doctors who may do us the honour to look into our pages.

